# On the structural formula of smectites: a review and new data on the influence of exchangeable cations

**DOI:** 10.1107/S1600576720016040

**Published:** 2021-02-01

**Authors:** Emilia García-Romero, Adrián Lorenzo, Andrea García-Vicente, Juan Morales, Javier García-Rivas, Mercedes Suárez

**Affiliations:** a Mineralogy and Petrology, Complutense University of Madrid, C/José Antonio Novais 12, Madrid 28040, Spain; b Geosciences Institute, Spanish Research Council and Complutense University of Madrid, C/José Antonio Novais 12, Madrid 28040, Spain; c Department of Geology, University of Salamanca, Plaza de la Merced s/n, Salamanca 37008, Spain; d Center for Nuclear Sciences and Technologies (C2TN), Instituto Superior Técnico, University of Lisbon, E.N. 10 (KM 139.7), Bobadela-LRS 2695-066, Portugal

**Keywords:** structural formula, homoionization, smectite, montmorillonite, beidellite, saponite, stevensite, kerolite

## Abstract

The understanding of the structural formula of smectite minerals is basic to predicting their physicochemical properties, which depend on the location of the cation substitutions within their 2:1 layer. This implies knowing the correct distribution and structural positions of the cations, which allows assigning the source of the layer charge of the tetrahedral or octahedral sheet, determining the total number of octahedral cations and, consequently, knowing the type of smectite. However, sometimes the structural formula obtained is not accurate. A key reason for the complexity of obtaining the correct structural formula is the presence of different exchangeable cations, especially Mg. Most smectites, to some extent, contain Mg^2+^ that can be on both octahedral and interlayer positions. This indeterminacy can lead to errors when constructing the structural formula. To estimate the correct position of the Mg^2+^ ions, that is their distribution over the octahedral and interlayer positions, it is necessary to substitute the interlayer Mg^2+^ and work with samples saturated with a known cation (homoionic samples). Seven smectites of the dioctahedral and trioctahedral types were homoionized with Ca^2+^, substituting the natural exchangeable cations. Several differences were found between the formulae obtained for the natural and Ca^2+^ homoionic samples. Both layer and interlayer charges increased, and the calculated numbers of octahedral cations in the homoionic samples were closer to four and six in the dioctahedral and trioctahedral smectites, respectively, with respect to the values calculated in the non-homoionic samples. This change was not limited to the octahedral sheet and interlayer, because the tetrahedral content also changed. For both dioctahedral and trioctahedral samples, the structural formulae improved considerably after homoionization of the samples, although higher accuracy was obtained the more magnesic and trioctahedral the smectites were. Additionally, the changes in the structural formulae sometimes resulted in changing the classification of the smectite.

## Abstract 2

The fit of the smectite structural formula is reviewed. In addition, a group of samples, both dioctahedral and trioctahedral, are studied, demonstrating the influence of interlaminar Mg that can lead to the erroneous classification of smectite if it is not considered.

## 1. Introduction   

Smectites have significant technical and industrial applications. In civil engineering, for instance, the behaviour of bentonites, which are natural rocks mainly composed of smectites, is crucial. Bentonites are used in the construction of antipollution barriers of different natures, such as highly radioactive deposits, landfills and contaminated soils. They are used in industry in diverse applications because of their absorbing and adsorbing properties (paint, paper and food industries, foundries, wastewater treatment, as additives in detergents or cat litter, or, because of their rheological properties, in drilling fluids). Thus, these applications derive from their unique physicochemical properties. Because of their very small particle size and microporosity, these minerals have a large specific surface area that, together with their layer charge and cation exchange capacity (CEC), gives them the ability to react with inorganic and organic polar reagents, mainly water (by hydration and dehydration). Additionally, they have swelling and rheological properties, and high plasticity. These properties are highly dependent on the amount of layer charge and on its location (Laird, 2006 ▸; Christidis *et al.*, 2006 ▸; Christidis, 2008 ▸), but also on the layer dimension because it determines the edge site properties (Delavernhe *et al.*, 2015 ▸). As an example, the thermal stability of mont­morillonites depends strongly on the distribution of octa­hedral cations over the *trans* and *cis* positions (Drits *et al.*, 1995 ▸; Emmerich *et al.*, 2009 ▸). Therefore, it is essential to know the crystal chemistry of smectites to address their industrial applications.

Smectite crystals are phyllosilicates with a 2:1 structure composed by stacking several layers of one octahedral sheet between two tetrahedral ones. Smectite layers have numerous isomorphic substitutions on both the tetrahedral (mainly Al^3+^, and secondarily Fe^3+^, instead of Si^4+^) and octahedral positions, as well as vacancies in the octahedral sheet, giving rise to a layer charge. This layer charge is compensated by cations (Mg^2+^, Ca^2+^, Na^+^, K^+^) in the interlayer space that link adjacent layers, are hydrated to different extents and may be exchanged with cations from an external solution. Importantly, the presence of these hydrated exchangeable cations is the reason behind their CEC. The net layer charge per unit formula (p.u.f.) in the smectite group ranges between 0.2 and 0.6, or between 0.4 and 1.2 per unit cell (p.u.c.) (Newman & Brown, 1987 ▸; Guggenheim *et al.*, 2006 ▸), although Emmerich *et al.* (2018 ▸) found some dioctahedral 2:1 layer silicates with a layer charge of 0.125 p.u.f. that are swellable. The weakly charged layers are held together by the electrostatic attraction of the interlayer cations. In addition to these, smectite and, in general, clay minerals absorb both anions and cations at the edges of the particles to compensate the broken bonds at the boundaries of the layers. Often, Mg^2+^ is one of the exchangeable cations, especially in the case of magnesic clays.

Different types of dioctahedral smectite have been recognized depending on the composition of the octahedral and tetrahedral sheets. Schultz (1969 ▸) distinguished different types of aluminous smectites and showed that the differences in their thermal properties can be related to their chemical composition: Wyoming, Otay, Tatatila and Chambers types are between the montmorillonite and beidellite end members, in a series of dioctahedral Al-rich smectites. However, regarding dioctahedral smectites, Brigatti & Poppi (1981 ▸) affirmed that ‘Chemical features do not confirm the continuity of the montmorillonite–beidellite series…A miscibility gap is also evident between nontronite and the other compositional ranges.’ Although most natural dioctahedral smectites have compositions between them, montmorillonite and beidellite themselves are extremely rare (Christidis, 2011 ▸). Dioctahedral smectite with a high octahedral iron content, where octahedral Fe^3+^ exceeds Al^3+^, is nontronite. Contrarily, if octahedral Al^3+^ exceeds Fe^3+^, the smectite is named as Fe^3+^-rich beidellite or Fe^3+^-rich montmorillonite (Guggenheim *et al.*, 2006 ▸). On the other hand, though the substitution of tetrahedral Si^4+^ for Fe^3+^ can be easily obtained in the laboratory, it appears to be rare or present in amounts below the detection limit of spectroscopic methods in natural samples (Finck *et al.*, 2019 ▸). Emmerich *et al.* (2009 ▸) added the configuration, *cis* or *trans*, as a new structural parameter required for the classification of dioctahedral smectites.

In trioctahedral smectites, if most octahedral sites are occupied by Mg^2+^ ions, the layer charge comes from the substitution of Si^4+^ by Al^3+^ in the tetrahedral sheet, and the mineral is saponite. Stevensite is a trioctahedral Mg-rich smectite with minor or without tetrahedral substitutions, having a deficit of cations in the octahedral sheet that leads to a low negative layer charge. Other species that have been described for the smectite group according to their crystallo-chemistry and structural formula are hectorite and swinefordite, which are trioctahedral smectites with Li^+^ as the octahedral cation, volkonskoite, which is dioctahedral and Cr^3+^ rich (Mackenzie, 1984 ▸; Khoury & Al-Zoubi, 2014 ▸), and rare ones such as sauconite, which is a dioctahedral Zn-bearing smectite (Ross, 1946 ▸; Balassone *et al.*, 2017 ▸). Newman & Brown (1987 ▸) compiled eight structural formulae of saponite with excess octahedral charge, and affirmed that ‘The net negative charge on the layers derives from Al for Si substitution in the tetrahedral sites, but this is partially compensated by substitution of trivalent cations into the octahedral sites.’ Similarly, Christidis (2011 ▸) asserted that ‘Saponite is different from the other smectites as part of the negative tetrahedral charge is balanced by substitution of octahedral Mg^2+^ by trivalent cations, Al^3+^ or Fe^3+^, *i.e.* the octahedral sheet often bears a positive charge. However, the tetrahedral charge due to substitution of Si^4+^ by Al^3+^ is much greater and outbalances any possible positive octahedral charge.’ However, Wilson (2013 ▸), in the compilation of 50 structural formulae of smectites of different composition and origin from different authors, reported that none of the studied smectites showed an excess of octahedral charge, although several would have Mg^2+^ as the interlayer cation.

The properties of smectites change not only with the magnitude of the layer charge but also with its distribution throughout the layer, with the exchangeable cations and with their hydration status (Güven, 1992 ▸; Laird, 1996 ▸, 1999 ▸; Meunier, 2006 ▸). The attractive force on the interlayer cations is more site specific for tetrahedral substitutions and reduces the number of hydration layers around the cations. This is because the Al^3+^ ionic substitution for Si^4+^ in the tetrahedral sheet causes an under-saturated valence in the three basal oxygens surrounding the Al^3+^ ions. Therefore, the negatively charged sites on the layer surface are point like. However, octahedral substitutions induce a more diffuse valence undersaturation for a large number of basal oxygens, because the charge imbalance diffuses through two more layers of ions in the structure. Therefore, the position of the cation substitution within the 2:1 structure influences the position of the negative charge on the surface of the layer (Güven, 1992 ▸; Meunier, 2006 ▸). This homogeneity has implications for the behaviour of the hydrated cations in the interlamellar space and on the surface of the smectites. Thus, for octahedral charged smectites such as montmorillonite, the negative charge is delocalized over surface oxygens so that only weak hydrogen bonds can form with interlayer water. For tetrahedral charged smectites such as beidellite and saponite, however, the charge is more localized and stronger hydrogen bonds can form between surface oxygens and interlayer water (Farmer, 1974 ▸). These different distributions of the interlayer charge, together with the different hydration statuses, lead to physicochemical properties that depend on the smectite type.

## 2. The structural formula of smectites   

The calculation of the structural formula is the only way to classify smectites according to their type and determine the amount and allocation of the charge that, together with the particle size and the *cis* or *trans* configuration, regulates most physicochemical properties. At present, however, despite the importance of having a reliable structural formula, it is nearly impossible to obtain the exact structural formula for a clay mineral, particularly for smectites. The first obstacle is to obtain a precise chemical composition avoiding the influence of impurities (*e.g.* SiO_2_ polymorphs, feldspars, zeolites, other clay minerals, carbonates, amorphous impurities *etc.*), since the composition is often obtained from whole-rock analyses and impurities of these types are commonly contained within the samples. There are some published papers in which the structural formulae were fitted from the results of chemical composition obtained by inductively coupled plasma emission spectroscopy (ICP-ES) or by X-ray fluorescence, either from raw samples or from the <2 µm fraction (*e.g. *Nadeau & Bain, 1986 ▸; Yeniyol, 2007 ▸, 2020 ▸). However, in most clayey samples, even the purest, small amounts of other minerals appear, not only in raw samples but also in the clay fraction in which there are frequently more than one phyllosilicate, and the composition of such impurities influences the calculated formula.

The interference from impurities can be avoided with electron microbeam techniques (Christidis & Dunham, 1993 ▸, 1997 ▸). There are two main techniques that allow one to obtain a quantitative chemical composition of isolated particles avoiding the influence of the impurities: electron microprobe analysis (EMPA) and analytical electron microscopy in transmission electron microscopy (AEM-TEM). EMPA has been used in several studies, like those of Ramseyer & Boles (1986 ▸) and Altaner & Grim (1990 ▸), although it is not used very often because it requires a perfectly polished and even sample surface for quantitative analysis, and as clayey samples are soft they usually have an irregular surface after polishing. However, TEM analyses of individual particles can be obtained from a representative powder portion of a sample, dispersed in ethanol or acetone, and deposited on a C-coated Au or Cu grid. Dispersion of the clay, frequently by sonication, allows the individual crystals or particles to disperse and deposit parallel to the grid surface. In these analyses the particles have to be sufficiently thin to be transparent to most of the primary X-rays produced by the incident beam and, therefore, X-ray absorption and fluorescence can be neglected (Lorimer *et al.*, 1976 ▸).

The structural fit from these techniques can be influenced by several technical limitations or by the intrinsic crystallo-chemical problems of smectite. Among the former, one obstacle is the impossibility of knowing the oxidation states of cations of the same elements like Mn, Ni and mainly Fe, which frequently appears as an octahedral cation as both Fe^2+^ and Fe^3+^, and sometimes as tetrahedral cations (Fe^3+^). Because the sedimentary, edaphic and weathering ambiences in which smectites normally appear are commonly associated with oxidizing conditions, Fe^3+^ is ordinarily considered, but this assumption can influence the octahedral occupancy and the distribution and amount of the charge layer. Kaufhold *et al.* (2019 ▸) also assumed all Fe as Fe^3+^ in a very detailed characterization of smectites from the Vetzia basin, and they pointed out that the tetrahedral charge values resulting from the structural formula calculation may vary depending on the Fe^2+^/Fe^3+^ ratio. García-Romero *et al.* (2019 ▸) studied the chemical composition of a wide group of almost-pure smectites by inductively coupled plasma mass spectrometry (ICP-MS) and determined the amount of Fe^2+^ by titration. They found that most samples only had Fe^3+^ and Fe^2+^ in a few samples with interstratified illite. On the other hand, the loss of light elements like Na and K is a significant problem; to minimize it, Nieto *et al.* (1996 ▸) tested the use of short counting times and compared the analyses obtained for different acquisition times ranging from 30 to 200 s, showing that shorter counting times gave improved reproducibility and normalized formula data.

If the data are obtained from EMPA or AEM at the thin edges of isolated particles, which provide data on domains having a diameter of a few nanometres, the structural formulae have to be the mean of a representative number of point analyses. This is because chemical and structural heterogeneity is typical among the individual crystals, as stated by Köster (1996 ▸) when he showed the structural and chemical variations in the different size fractions of the 2:1 layer minerals. Christidis & Dunham (1993 ▸) showed the wide variation in smectite composition among adjacent crystals found when different particles were analysed with electron microscopy methods, and they suggested that the average structural formulae do not provide enough indications about the variation range of the smectite population in individual samples. According to these authors, the source for this heterogeneity is related to (i) the proportion of tetrahedral charge relative to the octahedral charge, (ii) variable substitutions on octahedral positions, (iii) the relative abundances of exchangeable cations and (iv) the variation in the total layer charge.

In spite of these problems, the structural formulae of smectites obtained from microanalyses, whether from EMPA or from AEM, are probably the best approximation to the real formulae, and these methods have been used by several authors, including Ahn & Peacor (1986 ▸), Ramseyer & Boles (1986 ▸), Bouchet *et al.* (1988 ▸), Banfield & Eggleton (1990 ▸), Cheshire & Güven (2005 ▸), Christidis (2008 ▸), Cuadros *et al.* (2011 ▸), Berthonneau *et al.* (2014 ▸), Nieto *et al.* (2016 ▸), García-Romero *et al.* (2019 ▸) and Hoang-Minh *et al.* (2019 ▸). If the sample is not 100% monomineralic, the fit of the structural formula obtained by analytical electron microbeam techniques is nowadays considered the most accurate method. Probably, since they are not common techniques in clays laboratories, this is why there are relatively few articles in which the structural formulae of smectites are given and discussed, despite the tremendously rich research published in the field of smectites as Meunier (2005 ▸) pointed out.

To fit the structural formula of a phyllosilicate properly from the chemical composition it is necessary to fix one of the components. Because all tetrahedral and octahedral cations can be substituted, the number of negative charges is fixed as the sum of oxygen and hydroxyl groups (Lagaly & Weiss, 1976 ▸; Köster, 1977 ▸). In a second step, if the number of Si atoms is insufficient to complete the corresponding tetrahedral positions, some of the Al atoms are considered as tetrahedral. If there are still vacancies on the tetrahedral positions after using all the Al^3+^ ions, some of the Fe^3+^ ions are located there. The rest of the Al^3+^, Fe^3+^, Fe^2+^ and Mg^2+^ ions, and other elements such as Ni, Mn, Cr, Ti and Li, are allocated to octahedral positions. However, Ca^2+^, Na^+^ and K^+^ are considered as interlaminar cations, as is logical. In these four steps (defining the negative charge and the tetrahedral, octahedral and interlayer content) it is inevitable that there will be errors that, in the case of smectites, are not trivial.

Firstly, the assumption that all negative charge comes from oxygens and hydroxyl groups can be erroneous, because a part of the negative charge can derive from F^−^ substituting the hydroxyl groups of the octahedral sheet. Different amounts of F^−^ have been found in smectites, ranging from 0.02–0.45% for saponites from the Spanish Tajo Basin (Pozo *et al.*, 2014 ▸; García-Rivas *et al.*, 2018 ▸) to more than 5% for hectorite (Thomas *et al.*, 1977 ▸). A small amount of F^−^ can influence the final fit, though the main problem in having F^−^ is that if it is not possible to fix the negative charge, then the proportion of the cations cannot be normalized with respect to any other element. Other problems are related to the presence of non-exchangeable and non-structural cations (Kaufhold *et al.*, 2011 ▸), particle size (White & Zelazny, 1988 ▸), and the variable charges and local domains of different octahedral occupancy, as Wolters *et al.* (2009 ▸) pointed out.

A significant problem in fitting the structural formula of a smectite is the Mg allocation. Most smectites contain Mg^2+^ to some extent, and it is well known that this can be on both octahedral and interlayer positions. For instance, Christidis (2008 ▸) reported that ‘The most difficult question concerns allocation of Mg, which is assigned in octahedral sites’, although there are numerous reports for exchangeable Mg. Foster (1951 ▸) affirmed that ‘The presence of exchangeable magnesium in the montmorillonitic clays is more common than is generally recognized’, and Christidis (2011 ▸) remembered that ‘In analysis in which the smectite has not been rendered homoionic with an index cation other than Mg, allocation of Mg is usually a difficult task, because some of the Mg may be exchangeable’. Taking this into account, homoionization with a cation other than Mg^2+^ was done by several authors (*e.g. *Singh & Gilkes, 1991 ▸; Christidis & Dunham, 1993 ▸; Pozo & Casas, 1999 ▸; Cuevas *et al.*, 2003 ▸; Christidis & Mitsis, 2006 ▸; Fernández *et al.*, 2014 ▸; Sánchez-Roa *et al.*, 2016 ▸; Kaufhold *et al.*, 2019 ▸) prior to obtaining the structural formulae, to ensure that structural Mg is accounted for accurately. As mentioned before, Mg^2+^ is one of the main cations on the octahedral position in trioctahedral smectites, and frequently one of the interlayer cations in smectites. However, when the structural formulae are fitted, Mg^2+^ must be allocated on the octahedral position by default, unless different data are available.

When the octahedral occupancy is larger than 4 in dioctahedral smectites, some of the Mg might also be present in the interlayer, according to several authors. From this consideration, Herbert *et al.* (2004 ▸), Wilson *et al.* (2011 ▸), Nguyen-Thanh *et al.* (2017 ▸), Sánchez-Roa *et al.* (2018 ▸), Hoang-Minh *et al.* (2019 ▸) and Kadir *et al.* (2019 ▸), among others, allocate some of the Mg atoms as interlayer cations. According to them, if the sum exceeds 4 or 6 p.u.f., respectively, for dioctahedral and trioctahedral smectites, an amount of Mg equal to the difference in the number of octahedral cations should be allocated to the interlayer. This fitting criterion has also been followed by Elert *et al.* (2017 ▸, 2018 ▸), even for montmorillonite treated with a mixture of dry Mg-rich lime and water up to the plastic limit. Following this rule, only an approximation to the real structural formula is obtained, because it is not possible to be sure that the number of octahedral cations is exactly 4 or 6.

There has also been some research in which the structural formulae were fitted without considering the possible presence of Mg^2+^ as an interlayer cation in dioctahedral smectites (*e.g.* Cole, 1988 ▸; Altaner & Grim, 1990 ▸; Cheshire & Güven, 2005 ▸; Cuadros *et al.*, 2011 ▸; Vázquez *et al.*, 2014 ▸). The sum of the charges in the interlayer must balance the layer charge produced by the isomorphic substitutions on both tetrahedral and octahedral positions. In the absence of charge balance and in the presence of Mg^2+^, some of the Mg^2+^ should be assigned to the interlayer, even though it is impossible to determine the amount precisely. If the amount of exchangeable Mg^2+^ is high, the error could be high too. In fact, if Mg^2+^ is allocated to the octahedral position by default, and a part is in fact on interlayer positions, a structural formula fitted with all Mg^2+^ as octahedral cations will have a lower charge than the real sample. Consequently, not only the layer charge but also the smectite classification could be wrong.

To ensure the correct Mg^2+^ positions, that is to say, its real distribution on the octahedral and interlayer positions, it is necessary to exchange the interlayer Mg^2+^ and work with samples saturated with a known cation (homoionic samples). Homoionization also changes the cations adsorbed at the edges of the particles, and thus, the smaller the size of the particle, the higher the influence on the formula (Maes *et al.*, 1979 ▸; White & Zelazny, 1988 ▸).

Taking into account the factors discussed above, in this work the structural formulae of dioctahedral and trioctahedral smectite samples are calculated in order to demonstrate the importance of obtaining an accurate smectite layer charge, by assigning the interlayer cations precisely in the structural formula and, at the same time, evaluating the error when the formulae are calculated without previous homoionization of the samples. To achieve these aims, smectites have been studied in their natural form and after homoionization.

## 3. Materials and methodology   

### 3.1. Materials   

In the present work, seven smectite samples from different localities and different geological environments have been studied. They also have different chemical compositions and range from dioctahedral to trioctahedral smectites. Three samples (CAR1, CAR2 and LTBB) come from the Cabo de Gata volcanic region, located in the easternmost province of Andalusia in southern Spain. They are almost pure bentonitic deposits formed by the hydro­thermal alteration of the acid volcanic rocks (vesicular dark-coloured rhyodacites, glasses and weakly coloured ignimbrites, and tuffs). CAR1 and CAR2 come from the Cortijo de Archidona deposit, and LTBB from the Los Trancos deposit; both deposits have been studied previously (Reyes *et al.*, 1979 ▸, 1987 ▸; Fernández Soler, 1992 ▸; García-Romero & Huertas, 2017 ▸; García-Romero *et al.*, 2019 ▸). The WYO sample (Wyoming, USA) comes from the Repository of the Clay Minerals Society. Three samples (ESB6, RESQ and ROS) were collected at the Tajo Basin, located in the centre of the Iberian Peninsula. They are sedimentary clays belonging to the Pink Clays Unit (Martin de Vidales *et al.*, 1991 ▸; Pozo *et al.*, 1992 ▸; Cuevas *et al.*, 1993 ▸; Pozo & Casas, 1999 ▸; de Santiago Buey *et al.*, 2000 ▸; Cuevas *et al.*, 2003 ▸; García-Rivas *et al.*, 2018 ▸; García-Romero *et al.*, 2019 ▸). Tajo Basin is particularly interesting because it is one of the richest basins for Mg clays in the world, with high economic value. Samples ESB6 and RESQ were collected in a quarry in proximity to the locality of Esquivias (Madrid province, Spain), and ROS at the bottom of the Magán Hill, next to the village of Magán (Toledo province, Spain).

### 3.2. Methodology   

Smectite Ca saturation (homoionization with Ca^2+^) was done to replace the natural exchangeable cations by Ca^2+^. To make the cationic change, powdered samples were immersed in a 1 *M* CaCl_2_ solution, at room temperature, for three successive 24 h baths. Afterwards, the chloride solutions were removed, and the samples were washed with successive distilled water and centrifugation baths until chloride elimination was achieved. Chloride absence was confirmed with dilute AgNO_3_. Thus, the exchangeable cations that the smectites originally contained were replaced by Ca^2+^

Previous mineralogical characterization of the samples was carried out by means of X-ray diffraction (XRD) using a Siemens D500 diffractometer with Cu *K*α radiation and a graphite monochromator. The samples were measured as random powder specimens, and as air-dried, ethyl­ene glycol-solvated or heated (823 K for 2 h) oriented aggregates of the clay fraction (<2 µm). Powders were scanned in the range from 2 to 65° (2θ) at a scan speed of 0.05° 2θ in 3 s, and oriented aggregates from 2 to 30° (2θ), to determine the mineralogical compositions.

The chemical compositions were obtained by point analysis acquired by AEM-TEM. Samples for TEM observations were prepared by depositing a drop of diluted clay suspension onto a copper grid with a holey carbon film. Individual thin grains of the minerals were scattered onto the grids with the (001) planes parallel to the grid holder. In order to ensure the reproducibility of the data, the analyses were carried out at two different laboratories: at the Centro Nacional de Microscopía Electrónica (Spain) (CNME) and at the Centro de Instrumentación Científica, University of Granada, Spain (CIC). At the CNME two microscopes were used: a JEOL JEM 1400 microscope, with an acceleration voltage of 100 kV and 0.38 nm point-to-point resolution, and a JEOL 3000F field-emission microscope with an LaB_6_ filament at an acceleration voltage of 300 kV with 0.17 nm point to-point resolution. Both microscopes incorporate an energy-dispersive X-ray spectrometer (Oxford ISIS EDX, 136 eV resolution at 5.39 keV) analyser system, and an INCA microanalysis suite (Oxford Instruments), equipped with its own software for quantitative analysis. At the CIC, a Philips CM-20 scanning tunnelling electron microscope was used, operated at 200 kV [fitted with an ultrathin window and solid-state Si(Li) detector for energy-dispersive X-ray analysis]. The atomic percentages were calculated by the Cliff–Lorimer thin-film ratio criterion because AEM data were only collected from areas that could be clearly imaged by high-resolution transmission electron microscopy (HR-TEM). This restricts analysis to the very thin edges of the samples, thus satisfying the thin-film criterion of Lorimer *et al.* (1976 ▸). At the CIC, the validity of the *K* factors employed in the calculation of concentrations from the fluorescence intensities was checked using reference mineral samples according to Cliff & Lorimer (1975 ▸). Albite, biotite, spessartine, muscovite, olivine and titanite standards were used to obtain *K* factors for the transformation of intensity ratios to concentration following the procedures of Cliff & Lorimer (1975 ▸). Formulae were determined from atomic concentration ratios based on the number of oxygen atoms in the ideal formula. The structural formulae of the smectites were calculated on the basis of O_20_(OH)_4_. All the Fe present was considered as Fe^3+^ (owing to the limitation of the technique), but the possible existence of scarce Fe^2+^ cannot be excluded.

Particle morphology and textural relationships were established using HR-TEM at the CNME. The experimental conditions were optimized to avoid structural modification using a low beam intensity (<500 counts on the CCD camera) with an exposure time of 0.8 s to acquire the image. The samples were prepared through treatments to preserve the microtexture and avoid the collapse of the smectite interlayer space. These treatments are conducted in a sequence of successive steps where a small portion of the sample is placed in agar-agar to protect it from future stains. The sample must then be hydrated and the water progressively replaced by methanol; afterwards, the alcohol is replaced by Spurr resin, according to the methodology proposed by Tessier (1984 ▸) and Tessier & Pedro (1987 ▸). After polymerization of the resin, thin sections (50 nm) were cut by ultramicrotomy. This procedure minimizes dehydration during HR-TEM study and thus helps preserve the natural texture of the sample. The observations were performed using the JEOL 3000F field-emission microscope, equipped with a double-tilt sample holder (up to a maximum of ±23°) and a CCD camera for digital recording of the images.

## 4. Results and discussion   

**Figure 1:**
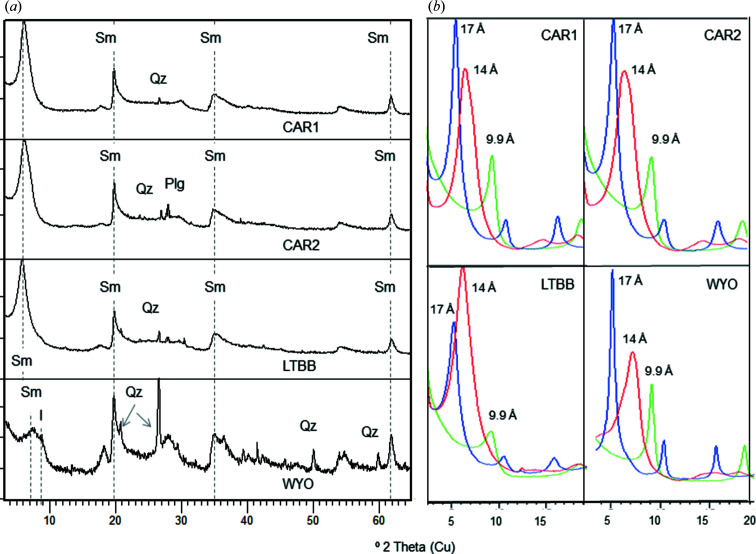
(*a*) Whole-rock powder XRD patterns for the dioctahedral samples studied. Plg denotes plagiclase, I illite, Qz quartz and Sm smectite. The dashed lines, from left to right, indicate the 001, 020, 131 and 060 smectite reflections, respectively. (*b*) XRD patterns of the oriented aggregates of the dioctahedral samples. Red denotes air-dried oriented aggregate, blue denotes ethyl­ene glycol-solvated oriented aggregate and green denotes heated (823 K) oriented aggregate.

**Figure 2:**
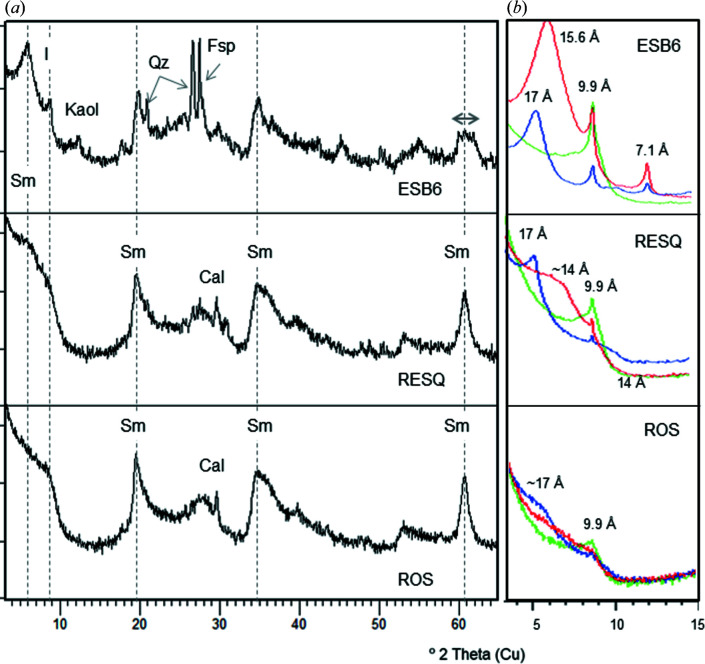
(*a*) Whole-rock powder XRD patterns for the trioctahedral samples studied. Fsp denotes feldspar, Cal Calcite, I illite, Kaol kaolinite, Qz quartz and Sm smectite. The dashed lines, from left to right, indicate the 001, 020, 131 and 060 smectite reflections, respectively. (*b*) XRD patterns of the oriented aggregates of the trioctahedral samples. Red denotes air-dried oriented aggregate, blue denotes ethyl­ene glycol-solvated oriented aggregate and green denotes heated (823 K) oriented aggregate.

The samples studied here are very pure and have a high proportion of smectite and small amounts of other minerals as impurities, mainly quartz, feldspars and/or calcite (Table 1 ▸, and Figs. 1 ▸ and 2 ▸). Four of the seven samples studied (CAR1, CAR2, LTBB and WYO) are rich in dioctahedral smectites, as shown by their 060 reflection at 0.149 nm (2θ = 61.9°), and the other three (ESB6, ROS and RESQ) are trioctahedral (060 reflection at 0.152 nm, 2θ = 60.7°). The 060 reflection of the ESB6 sample is wider than that of the rest (Fig. 2 ▸), indicating a mixture of di- and trioctahedral phyllosilicates. Quartz is the most frequent impurity, though it appears in very small amounts in the WYO and CAR1 samples, and as traces in ESB6, CAR2 and LTBB. ESQ6 also contains illite, kaolinite and feldspars. ROS and RESQ contain a very small amount of calcite. Three of the dioctahedral samples (CAR1, CAR2 and LTBB) have good crystallinity, as evidenced by their narrow 001 reflection and the relative intensities of the smectite reflections. At the other extreme, ROS and RESQ have high defects of staking, as can be seen by the absence of a clear 001 reflection, which rather appears as a very broad band in their XRD patterns. The smectitic nature of this sample is demonstrated by its swelling after ethyl­ene glycol solvation (Fig. 2 ▸).

**Figure 3:**
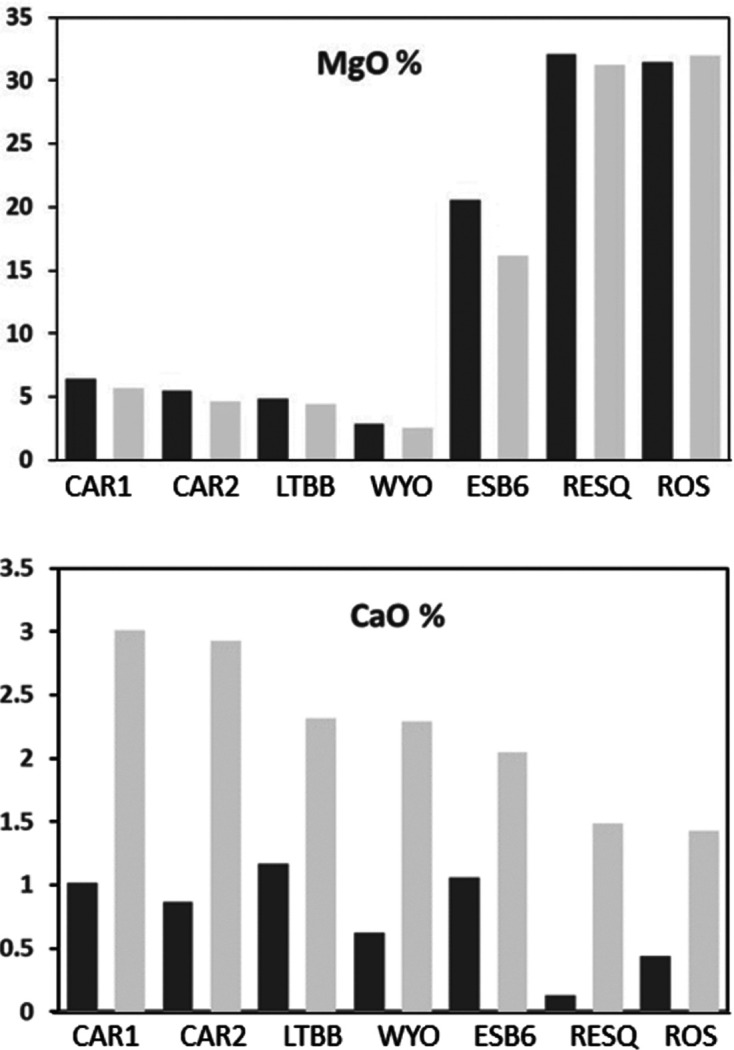
MgO and CaO content (in %) of the natural and Ca^2+^ homoionic samples. Note that MgO decreases and CaO increases after homoionization. Dark-grey bars show results before homoionization and light-grey bars show results after homoionization.

All samples were analysed both before and after their homoionization with Ca^2+^. The mean contents of the major oxides are reported in Table 2 ▸, and the structural formulae calculated from the mean chemical compositions of both natural and homoionized smectites are shown in Table 3 ▸. The SiO_2_ content ranges between 60.55% (standard deviation SD = 2.28) (ESB6) and 67.89% (SD = 0.92) (CAR2), and it is slightly higher for dioctahedral smectites than for triocta­hedral ones. The dioctahedral samples are the richest in Al_2_O_3_ because Al^3+^ is their main octahedral cation, although all of them also contain Fe_2_O_3_ and MgO, while the three trioctahedral samples are the richest in MgO, as expected. When comparing natural and Ca homoionic samples, a difference can be observed in all oxides. As is logical, the content of CaO increases in the homoionic samples, confirming the successful exchange of cations. It is highlighted that the content of MgO decreases after cation exchange in all samples except for ROS, where this oxide slightly increases (Fig. 3 ▸).

Regarding the structural formulae of the dioctahedral samples, CAR1 and CAR2 come from different points of the same deposit (Cortijo de Archidona, Spain) and their chemical compositions are similar (Table 2 ▸). However, because they are natural samples they have small compositional differences that lead to a different distribution of charges. These small differences in chemical composition imply a difference in their structural formulae and classification: because its tetrahedral charge is higher than its octa­hedral one, CAR1 has to be classified as a low-charge beidellite, whereas CAR2 does not have tetrahedral charge and is classified as a low-charge montmorillonite. Samples from the Cortijo de Archidona deposit were classified as montmorillonites in previous work (Caballero *et al.*, 2005 ▸; García-Romero & Huertas, 2017 ▸). In both cases, the layer charge is very low, just above or below the lower smectite limit. This is especially true for CAR2, in which this parameter is −0.30 p.u.c. After fitting, the natural sample from Los Trancos (LTBB) is classified as a low-charge beidellite, with a layer charge of −0.29, below the theoretical limit for smectites (−0.4 p.u.c). In previous work, this sample was also classified as a montmorillonite (Reyes *et al.*, 1979 ▸; García-Romero & Huertas, 2017 ▸).

**Figure 4:**
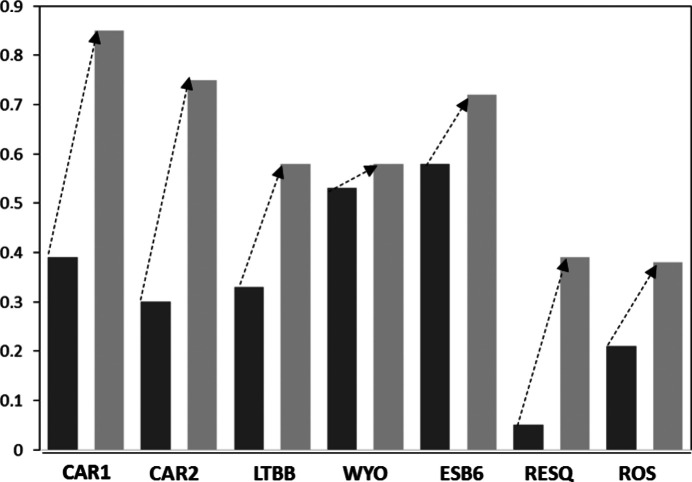
Differences in the interlayer charge of the samples after Ca^2+^ homoionization. Dark-grey bars show results before homoionization and light-grey bars show results after homoionization. Note that the interlayer charge increases in all samples.

The formulae fitted from the mean chemical compositions of these three samples after homoionization correspond to montmorillonites. The small variations in the MgO content (<1%) in Ca smectites imply a change in the structural formulae with respect to the natural samples. The calculated layer charge increases for the non-homoionic samples in the three cases (Table 3 ▸ and Fig. 4 ▸).

**Figure 5:**
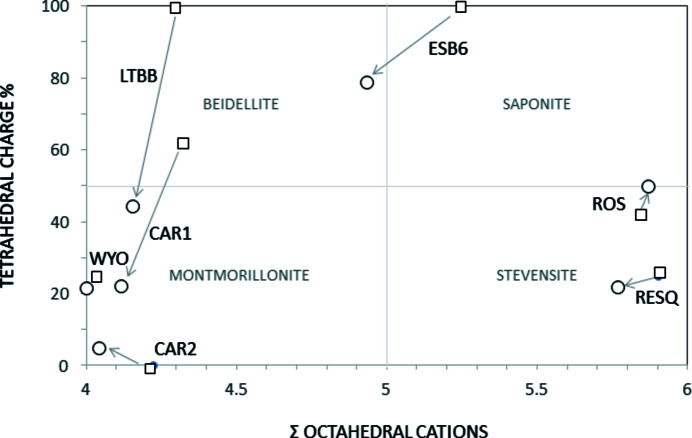
Octahedral cation numbers as a function of the octahedral charge and classification of the smectites according to the plotting area. Squares are natural samples and circles are Ca homoionized samples.

The classification of samples CAR1 and LTBB changes from low-charge beidellite to montmorillonite (Fig. 5 ▸) because despite having more charge, most of it is now located on the octahedral sheet. CAR2 changes the montmorillonite subtype, according to the classifications of Schultz (1969 ▸) and Emmerich *et al.* (2009 ▸), because the homoionic sample has a small tetrahedral charge, while the natural sample does not. The difference in the structural formulae is not very large when comparing the numbers of tetrahedral and octahedral cations of each sample, which change by a maximum of 0.1 p.u.c. However, the structural formulae of the homoionic samples fit accurately, and better than the natural samples, because the layer charge values are in the smectite range in the three cases (−0.88, −0.73 and −0.58 for CAR1 Ca, CAR2 Ca and LTBB Ca, respectively).

Cuadros *et al.* (1994 ▸) reported that, in a group of smectites from hydro­thermal alteration of a very homogeneous volcanic tuff of acid composition from the Cabo de Gata deposits, similar to CAR1, CAR2 and LTBB, some of the samples chemically characterized as beidellite behaved as mont­morillonites in the Li test (Greene-Kelly, 1953 ▸). This could be related to an erroneous structural formula deriving from the presence of Mg^2+^ as the exchangeable cation, as in the case of CAR1 and LTBB. In the same way, the excess of positive octahedral charge reported by Newman & Brown (1987 ▸) and Christidis (2011 ▸) could be caused by an erroneous assignment of Mg^2+^ to the octahedral layer instead of the interlayer.

The WYO sample, however, only shows some slight changes in the structural formula after homoionization due to the small amount of MgO in the natural sample, which leads to a small difference in its chemical composition after homoionization with Ca^2+^.

All trioctahedral samples were collected at the Tajo Basin (Spain) and they belong to the same unit, the Pink Clays Unit, so their chemical compositions should be similar. These clays have been studied by different authors and have been characterized as stevensite (Cuevas *et al.*, 1993 ▸, 2003 ▸; de Santiago Buey *et al.*, 2000 ▸; García-Rivas *et al.*, 2018 ▸), kerolite (Pozo & Casas, 1999 ▸) or interstratified kerolite/stevensite (Martin de Vidales *et al.*, 1991 ▸; Pozo *et al.*, 1992 ▸, 1999 ▸; Pozo & Casas, 1999 ▸; Clauer *et al.*, 2012 ▸), and as a fine-grained interstratification of turbostratic talc and saponite (Steudel *et al.*, 2017 ▸). The lack of agreement on the classification of Pink Clays is easily understood by considering the chemical compositions and structural formulae obtained from our natural samples.

**Figure 6:**
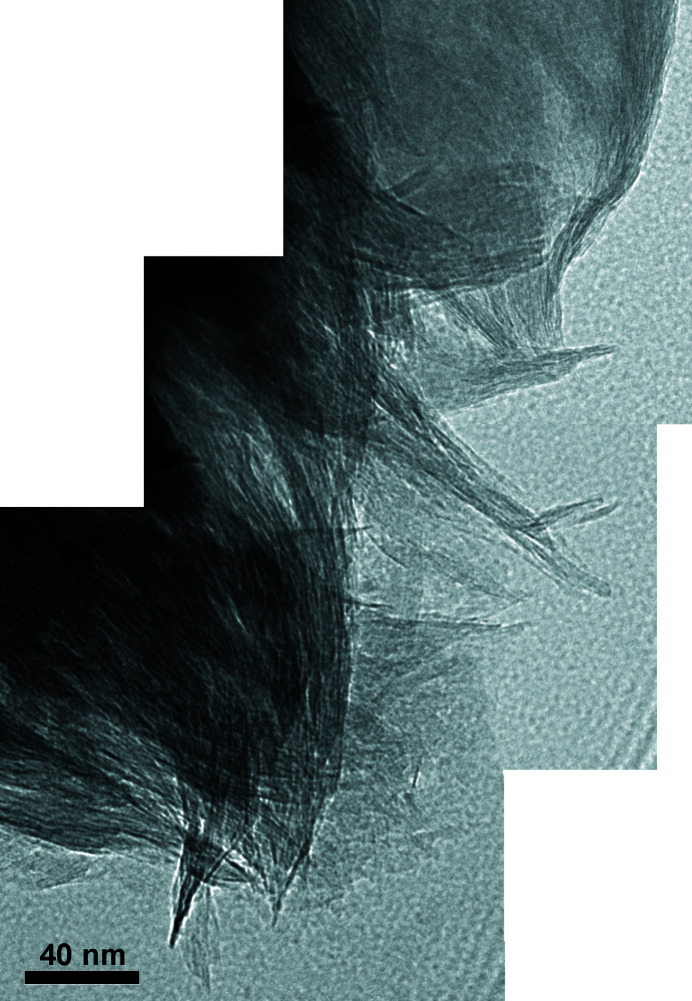
HR-TEM images of sample RESQ showing the particle edge composed of small subunits that form the larger ones. They have a very small particle size and numerous stacking faults and edge dislocations. Note the characteristic smectite morphological features with their common sigmoidal appearance. The subunits are thicker in their central portions, with tapered margins and curved cross sections.

As seen in Table 3 ▸, samples RESQ and ROS present some problems. RESQ could be classified as a kerolite, because it does not have interlayer charge. Consequently, the classification as kerolite or interstratified kerolite/stevensite made by several authors could be correct. However, in the homoionic sample the layer charge increases and the sample can be classified as a low-charge stevensite. In the case of ROS, its layer charge is too low for a smectite (−0.19) and its octa­hedral and tetrahedral charges are close, so this sample should be classified as kerolite, in agreement with other authors who classified samples from the same unit as kerolite or interstratified kerolite/stevensite. However, the classification as kerolite does not agree with the properties of this clay, namely its partial swelling ability (Fig. 2 ▸) and its high specific surface area of 392 m^2^ g^−1^ (de Santiago Buey *et al.*, 2000 ▸). The HR-TEM photographs (Fig. 6 ▸) show the characteristic morphological features of RESQ, displaying the edges of particles composed of small subunits that form the larger particles. Both have the common sigmoidal appearance and parallel lattice planes. The subunits are thicker in their central portions, with tapered margins and curved cross sections. They have a very small particle size and numerous stacking faults and edge dislocations, as described by de Santiago Buey *et al.* (2000 ▸) and García-Romero & Suárez (2018 ▸).

**Figure 7:**
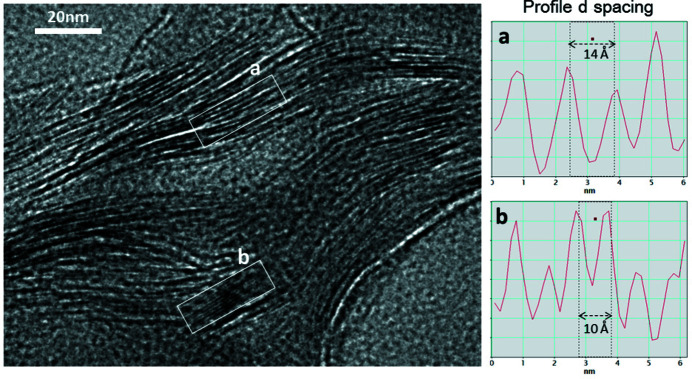
(Left) An HR-TEM image showing the small areas with 10 Å *d* spacings (labelled b) included in the general ∼14 Å *d* spacings (labelled a) of smectite (ESB6 sample). Note the very small 10 Å areas commonly show different features since they have a straight and regular grid with 10 Å spacing, free of dislocations, stacking faults and edge dislocations.

After homoionization, the formulae of trioctahedral smectites change (Table 3 ▸) and their layer charge increases (Fig. 4 ▸). RESQ and ROS change from kerolite to stevensite in the homoionic samples, although with very low charge. ESB6 Ca has 4.93 octahedral cations p.u.c. and tetrahedral charge, and it should be classified as intermediate between beidellite and saponite (Fig. 5 ▸). However, it is necessary to take into account that the 060 reflection of the ESB6 sample is wide, as has already been indicated above, which means that it is a mixture of di- and trioctahedral phyllosilicates. The sample contains discrete illite and a trioctahedral smectite (saponite) with minor proportions of dioctahedral mica layers interstratified (Fig. 7 ▸) or small clusters of illite included in the smectite particles, in agreement with Hoang-Minh *et al.* (2019 ▸). This explains the minor proportions of interlayer K^+^ that remain in its structural formula after homoionization, when the point analyses on smectite particles are obtained. The structural formulae obtained after homoionization of these trioctahedral smectites are more accurate because the uncertainty in the position of Mg^2+^ has been avoided. Fig. 7 ▸ shows small areas with 10 Å spacings included in the general 14 Å spacing smectite. Small 10 Å areas have been observed randomly distributed along the ESB6 sample. Those 10 Å areas commonly display different features since they have a straight and regular grid with 10 Å spacing, free of dislocations, stacking faults and edge dislocations. The presence of these 10 Å micaceous layers leads to a higher tetrahedral charge in the mean value for the particle

The study of these trioctahedral smectites, mainly of the very complex samples from the Pink Clays Unit, prior to and after homoionization, shows the importance of the correct allocation of octahedral Mg^2+^. As for dioctahedral smectites, the layer charge of the exchanged trioctahedral smectites is higher. There are other cases in the literature in which the structural formulae for trioctahedral 2:1 minerals, without removing the interlayer Mg^2+^, fitted for minerals of very low charge (Yeniyol, 2007 ▸). Some of these results could be partially influenced by a lack of knowledge of the octahedral and interlayer Mg^2+^ distribution.

Because fitting the structural formulae consists of distributing cations on the octahedral and tetrahedral positions, homoionization has an effect not only on the positions occupied by Mg^2+^ but also on the full distribution of the cations, as has already been indicated by some authors [such as Christidis (2008 ▸), and references therein]. Because the charge of the layer must be between 0.4 and 1.2 for O_20_(OH)_4_ (Guggenheim *et al.*, 2006 ▸), an increase in the interlayer charge improves the fitted structural formula considerably, and it reveals a more accurate crystal chemistry of smectites, decreasing their chemical artefacts and, in some cases, modifying their classification (Table 3 ▸ and Fig. 4 ▸). This change is a consequence of the Mg^2+^ which was wrongly assigned to the octahedral position, and our data show that Mg^2+^ is more common than generally recognized in montmorillonitic clays. The changes detected are only a response to the recalculation of the relative proportions of the cations with the new formulae. Ca^2+^ is the only cation expected in the interlayer position in the analysis of homoionic smectites.

Finally, Emmerich *et al.* (2009 ▸) concluded ‘The smectite structure reveals five features that allow an unambiguous description of a sample: 1) identification as either a dioctahedral or a trioctahedral smectite; 2) layer charge; 3) charge distribution between tetrahedral and octahedral sheets; 4) cation distribution within the octahedral sheet and 5) Fe content. In addition, the nature of interlayer cations should be given as they influence certain properties of montmorillonites.’ To analyse these structural parameters, the structural formula must be fitted for a sample with no interlayer Mg^2+^. Currently, the only way to do this is to perform the chemical analysis after homoionization with a different cation. This ensures that (i) the assignation of the cations to the tetrahedral and octahedral positions, and therefore the distribution of the layer’s charge, is correct, and (ii) the structural parameters can be related to the physicochemical properties.

## 5. Final remarks   

Homoionization with Ca^2+^ produces an important difference in fitting the structural formulae, not only for trioctahedral and Mg-rich smectites, which is expected, but also for diocta­hedral smectites.

For both dioctahedral and trioctahedral samples, the interlayer charge increases notably in the homoionic samples because the octahedral charge increases. Additionally, changes are observed in the tetrahedral content and charge. In the homoionic samples, the number of octahedral cations is closer to four and six in dioctahedral and trioctahedral smectites, respectively, with respect to the natural samples. Overall, a better fit of the formulae is obtained for the Ca^2+^ homoionic smectites. Furthermore, the classification of the smectite type changes for several samples after homoionization, which eliminates the interlayer Mg.

Because the structural formulae obtained after homoionization of the samples are more accurate, it can be concluded that homoionization improves the structural formulae fitting for both dioctahedral and trioctahedral smectites. In this context, homoionization is strongly recommended routine to avoid mistakes, especially when the structural formulae, structural parameters and, in general, crystal-chemical data must be related to the physicochemical properties of the samples for practical applications.

## Figures and Tables

**Table 1:** Main data for the studied samples, including labels, location and mineralogical compositions (from XRD data) of impurities that appear with the smectite The order of impurities is related to their abundance, starting with the most abundant. Minerals in parentheses are ≤5% in weight, and minerals indicated with an asterisk (*) are present at trace level.

Quarry (location)	Label	Impurities
Cortijo de Archidona (Cabo de Gata, Spain)	CAR1	(Quartz)
	CAR2	(Plagioclase, Quartz)
Los Trancos (Cabo de Gata, Spain)	LTBB	(Quartz, Plagioclase*)
Crook County (Wyoming, USA)	WYO	Quartz, (Illite)
Esquivias (Tajo Basin, Spain)	ESB6	Illite, (Quartz, Kaolinite, Feldspar)
Magán Hill (Tajo Basin, Spain)	RESQ	Calcite*
	ROS	Calcite*

**Table 2:** Mean chemical compositions of natural and homoionic samples obtained by point analysis in AEM SD denotes standard deviation.

Sample		SiO_2_	Al_2_O_3_	Fe_2_O_3_	MgO	CaO	NaO	K_2_O
CAR1	Mean *n*: 42	65.58	22.45	3.83	6.38	1.02	0.26	0.48
	SD	1.27	1.16	1.70	0.94	0.31	0.41	0.40
CAR1 Ca	Mean *n*: 26	65.94	21.68	3.14	5.72	3.01	0.20	0.28
	SD	0.48	0.38	0.75	0.42	0.37	0.22	0.15
CAR2	Mean *n*: 27	67.89	19.97	5.26	5.53	0.87		0.55
	SD	0.92	1.82	2.27	0.49	0.21		0.53
CAR2 Ca	Mean *n*: 24	67.85	22.02	2.54	4.61	2.93		0.05
	SD	1.07	0.79	0.42	0.36	0.29		0.22
LTBB	Mean *n*: 28	65.55	25.58	2.36	4.86	1.17	0.09	0.08
	SD	2.44	2.89	1.17	1.17	0.94	1.07	0.21
LTBB Ca	Mean *n*: 20	66.55	24.89	1.86	4.38	2.32		
	SD	1.33	2.19	0.74	1.09	0.96		
WYO	Mean *n*: 46	66.67	23.54	4.42	2.90	0.62	1.12	0.73
	SD	1.68	1.14	1.29	0.51	0.69	1.03	0.94
WYO Ca	Mean *n*: 31	66.94	23.67	4.49	2.56	2.29	0.02	
	SD	1.29	0.62	0.79	0.41	0.48	0.09	
ESB6	Mean *n*: 33	60.55	12.22	3.70	20.60	1.06	0.07	1.80
	SD	2.28	4.43	1.86	4.86	1.70	0.16	1.24
ESB6 Ca	Mean *n*: 19	60.94	14.80	4.80	16.21	2.05		1.19
	SD	2.60	7.34	2.27	5.66	1.00		1.15
RESQ	Mean *n*: 97	66.38	1.02	0.25	32.12	0.13	0.02	0.07
	SD	2.65	1.61	0.50	2.58	0.32	0.04	0.49
RESQ Ca	Mean *n*: 62	65.76	1.00	0.42	31.22	1.49		0.03
	SD	3.46	0.67	0.89	4.30	1.37		0.31
ROS	Mean *n*: 50	65.64	1.50	0.57	31.46	0.44	0.29	0.11
	SD	1.62	1.24	0.63	2.40	0.27	0.72	0.22
ROS Ca	Mean *n*: 50	64.27	1.20	1.02	32.02	1.43	0.02	0.02
	SD	1.31	0.49	2.05	2.28	0.32	0.17	0.07

**Table 3:** Structural formulae [for O_20_(OH^−^)_4_] and parameters of the natural and homoionic samples obtained from the mean chemical compositions So is the number of octahedral cations, St the number of tetrahedral cations, CT the tetrahedral charge, CO the octahedral charge and CI the interlayer charge.

	Si	Al^IV^	Fe^3+^	Al^VI^	Fe^3+^	Mg	Ca	Na	K	So	St	CT	CO	CT+CO	CI	Classification
CAR1	7.74	0.26		2.86	0.34	1.12	0.13	0.06	0.07	4.32	8.00	−0.26	−0.16	−0.42	0.39	Low-charge beidellite
CAR1 Ca	7.80	0.20		2.82	0.28	1.01	0.38	0.05	0.04	4.11	8.00	−0.20	−0.68	−0.88	0.85	Montmorillonite
CAR2	8.01			2.78	0.47	0.97	0.11		0.08	4.22	8.01	0.00	−0.31	−0.30	0.30	Low-charge montmorillonite
CAR2 Ca	7.96	0.04		3.01	0.22	0.81	0.37		0.01	4.04	8.00	−0.04	−0.69	−0.73	0.75	Montmorillonite
LTBB	7.69	0.31		3.23	0.21	0.85	0.15	0.02	0.01	4.29	8.00	−0.31	0.02	−0.29	0.33	Low-charge beidellite
LTBB Ca	7.74	0.26		3.21	0.17	0.77	0.29			4.15	8.00	−0.26	−0.32	−0.58	0.58	Montmorillonite
WYO	7.86	0.14		3.13	0.39	0.51	0.08	0.26	0.11	4.03	8.00	−0.14	−0.42	−0.56	0.53	Montmorillonite
WYO Ca	7.87	0.13		3.15	0.40	0.45	0.29			4.00	8.00	−0.13	−0.45	−0.58	0.58	Montmorillonite
ESB6	7.40	0.60		1.16	0.34	3.75	0.14	0.02	0.28	5.25	8.00	−0.60	0.00	−0.60	0.58	Saponite
ESB6 Ca	7.42	0.58		1.55	0.44	2.94	0.27		0.18	4.93	8.00	−0.58	−0.15	−0.73	0.72	Saponite
RESQ	7.98	0.02		0.12	0.02	5.76	0.02		0.01	5.90	8.00	−0.02	−0.06	−0.08	0.05	Kerolite
RESQ Ca	7.92	0.08		0.13	0.04	5.60	0.19		0.01	5.77	8.00	−0.08	−0.29	−0.37	0.39	Low-charge stevensite
ROS	7.92	0.08		0.14	0.05	5.66	0.06	0.07	0.02	5.85	8.00	−0.08	−0.11	−0.19	0.21	Kerolite
ROS Ca	7.81	0.17	0.02		0.07	5.80	0.19			5.87	8.00	−0.19	−0.19	−0.38	0.38	Low-charge saponite–stevensite
